# SHAFE Mapping on Social Innovation Ecosystems

**DOI:** 10.3390/ijerph20010118

**Published:** 2022-12-22

**Authors:** Carina Dantas, Juliana Louceiro, Joana Vieira, Willeke van Staalduinen, Oscar Zanutto, Karolina Mackiewicz

**Affiliations:** 1 SHINE 2Europe, 3030-163 Coimbra, Portugal; 2 AFEdemy—Academy on Age-Friendly Environments in Europe, 2806 ED Gouda, The Netherlands; 3 Istituto per Servizi di Ricovero ed Assistenza agli Anziani, 31100 Treviso, Italy; 4 ECHAlliance—European Connected Health Alliance, 13A Ballyhoy Avenue, 5 Dublin, Ireland

**Keywords:** social innovation, SHAFE, network, ecosystems, costs benefit and avoided costs, urban complex ecosystems, long-term sustainable future

## Abstract

There have been several initiatives aiming to promote innovation and support stakeholders to increase investments in relevant societal areas connected to Smart Healthy Age-Friendly Environments—SHAFE. However, their impact usually runs shorter than desirable in the mid- and long-term due to the difficulty to identify, map, and connect stakeholders in the different European and world countries that are willing to work for the practical implementation of social innovation around SHAFE. This mapping and connection can contribute to increase awareness of innovation actors on social innovation concepts and, if well disseminated, may also leverage the creation of alliances and synergies between different stakeholders within ecosystems and between ecosystems. Understanding what relevant practices exist, how they are funded, and how they involve citizens and organisations is also key to ensure that business actors have access to social innovation and entrepreneurial knowledge, which is key for future sustainable societal change. The present study developed and implemented a survey replied by 61 organisations from 28 different countries. The results showed relevant inputs regarding different cultural and societal perceptions, including diverse end-user organisations, and will, thus, facilitate multistakeholder engagement, public awareness, and the overall upscaling of social innovation on SHAFE.

## 1. Short Glossary

SHAFE: Smart Healthy Age-Friendly Environments is a conceptual and policy approach referring to smart, adaptable, and inclusive environments, products, and services that can help improve and support independence and wellbeing throughout the course of life, regardless of age, gender, disabilities, cultural differences, and personal choices.

Ecosystem: a geographically focused, permanent, multi-stakeholder partnership committed to working together to implement innovative solutions that improve the quality of health and wellbeing of citizens, consisting of citizens, public authorities, businesses, NGOs, and research.

Quadruple helix innovation framework: describes the university–industry–government–public interactions within a knowledge economy, with implications for smart co-evolution of regional collaborations.

## 2. Introduction

### 2.1. About Social Innovation

The concept of social innovation is drawn on multiple layers and encompasses multiple elements, such as the transformation of governance arrangements, tools, and participation forms; new relationships within society and its different actors; systemic adaptation at the social level. One key aspect is grounded on the role of citizens and their participatory role, as they are the ones in the position to evolve “initiatives from a localised level to a macro-level” [1]. In a domain such as Smart Healthy Age-Friendly Environments (SHAFE), social innovation is useful to provide practical insights into how implementation of new technologies and services can be enhanced in societal systems pathways and practice.

Social Innovation processes can be useful to understand the role of citizens (or specific target groups) in societal transformation. Empowered citizens will be able to be part of the implementation pathways not as service recipients but as “the leaders and ultimately the owners of (for example) health interventions and programmes” [2].

Social innovation can best be understood as innovation in social relations, in power dynamics, and in governance transformations, and may include institutional and systems transformations. It has been applied in health research, within multiple topics, mainly from an instrumental and technocratic point of view, to foster greater patient and beneficiary participation in health programmes. However, to achieve this degree of participation, a critical view on the structures of power needs to be undertaken and public authorities are essential to be on board with such initiatives. If the quadruple helix [3] of stakeholders in one community is aligned and willing to open their minds and hearts to new experiments and participatory initiatives, there is room for “shifting the power dynamics” thus creating “new avenues for involvement and participation” [4].

Social Innovation provides a framework for understanding systems innovation, but goes well beyond the existing premises of functional efficiency or incremental improvement. The concept of social innovation launched the idea of creating communities from the perspective of people who actively voice their ideas and solutions, especially those that emerge at the grassroots level, thus enhancing equity and empowerment.

Social innovation is inherently interdisciplinary and intersectoral, bringing added value for policymaking as it emphasises the context in the implementation. It usually discards any solutions of “one-size fits all”, accounting for uniqueness and the specific conditions of each implementation site. Therefore, a mapping of existing initiatives is helpful to understand current practices and potentially benchmark them, adding value to new communities wishing to embrace such challenges. From the gathering of initiatives in different countries and networks, it will be possible to create future synergies and promote a group of early adopters that are able to progress on SHAFE implementation and upscale.

However, possibly due to its holism and multidisciplinarity, “the dynamics of social innovation processes are of a complex nature and an underdeveloped research field” [5]. Concepts such as Responsible Research and Innovation, Value-Sensitive Design, and Social Innovation are seen by many “as phenomena posing an abstract ideal that still lack practical evidence to support its claims and assumptions.”

The study of social innovation has involved qualitative, quantitative, and mixed methods, and has focused on analyzing innovation at the micro- or macro-level, understanding and measuring its impact, uncovering the systemic relations behind it, or other features [6]. The last few years brought several new methods to the social sciences and social innovation such as “linear models, mixed methods, systems frameworks, machine learning, and new approaches to fieldwork” [7].

However, social innovation is not always framed within strict theoretical models and it often needs to be given the possibility of open social experiments, in practical contexts and with multiple stakeholders without predetermined goals or plans. This is the case, for example, of the social labs methodology, “where experts and stakeholders join together to initiate actions focused on tackling challenges without being constrained by predetermined project plans, lists of deliverables, and—most importantly in this context—without knowing exactly how to proceed” [8].

### 2.2. Social Innovation Networks in NET4Age-Friendly

#### 2.2.1. SHAFE and NET4Age-Friendly

The COST Action 19136, NET4Age-Friendly—International Interdisciplinary Network on Smart Healthy Age-Friendly Environments [9]—aims to promote the creation and implementation of smart, healthy environments, for different ages and throughout the life course, with a special focus on overcoming fragmentation and critical gaps at both conceptual and pragmatic innovation to address European research and policy challenges. Given its policy focus and the strong roots to social innovation methods, it has grown based on a quadruple helix approach with resource to participatory methods. The quadruple helix model of innovation identifies “four major actors in the innovation system: science, policy, industry, and society” [9]. The SHAFE network and the COST Action NET4Age-Friendly consider this as the cornerstone of the development of the network and its work, mostly aimed at the sharing and transfer of practical successful practices.

This international network, with nearly 500 members from 51 countries (as of October 2022), proposes a holistic approach to optimise social and physical environments, supported by digital tools and services, promoting healthy and active living, equity in access to service, and active participation in society. SHAFE [10], an acronym for Smart Healthy Age-Friendly Environments, follows the United Nations Sustainable Development Goals (in particular, Objectives 3 and 11), stating that sustainable environments for all ages represent the basis for ensuring a better future for the entire population and addressing most of the growing issues of the ageing population.

It started its route as a Thematic Network, approved by the European Commission, to draw policymakers’, organisations’, and citizens’ attention to the need of better alignment between health, social care, built environments, and ICT, both in policy and funding. To this aim, the Network delivered a Joint Statement and a Framing Paper in December 2018 to the European Commission and Member States. After this, it evolved to a European Stakeholders Network, with over 170 partner organisations and more recently, in 2021, to the SHAFE Foundation. This Foundation aims to develop, promote, fund, and award fundamental and applied research, as well as actions and activities related to policymaking and skills development, enabling the promotion and implementation of SHAFE at all levels [11]

The challenges of different sectors, such as building environments and urban planning, the digital revolution, and the need for more efficient and sustainable health and social care, are closely interlinked and essential for increasing the quality of life and wellbeing of citizens and their communities. SHAFE responds to these challenges by promoting the creation and implementation of smart, healthy, and inclusive environments for all generations that enable them to learn, grow, work, socialise, and enjoy a healthier life, benefiting from the use of digital innovations, accessibility solutions, and adaptable support models in the European context.

The main approach of NET4Age-Friendly is to implement SHAFE through the establishment of new local or regional ecosystems or by expanding existing ones in each European COST country involved, to work on health and wellbeing in an age-friendly digital world. These ecosystems include citizens, public authorities, academia and research, industry, and NGOs and are supported by five thematic Working Groups: User-centred inclusive design in age-friendly environments and communities; Integrated health and well-being pathways; Digital solutions and large-scale sustainable implementation; Policy development, funding forecast and cost-benefit evaluations; Reference Framework.

#### 2.2.2. ECHAlliance Ecosystems

ECHAlliance Ecosystems bring together a permanent community of stakeholders to develop a joint health agenda, aiming to address and find common solutions to regional health challenges. Through Ecosystems, needs and solutions match and stakeholders break down silos, transform healthcare delivery, and create economic growth. The key benefit of working together in an Ecosystem is the multiplier effect of collaborating in an International Network of Ecosystems, as an intelligence network connecting the dots in digital health. The ECHAlliance have built a network so far of more than 70 Ecosystems across the globe.

#### 2.2.3. Faber

The Housing and Health Thematic Innovation Ecosystem connects and works on specific areas: Smart Buildings; Green Approach to Building and Design; Innovative Social Models; Finance, Economic, and Sustainability Governance. It is led by ISRAA, reaching out to more than 2000 experts, public bodies, and care providers EU-wide, adding the ISRAA’s European Department, that is, Faber Fabbrica Europa [12], with 1000 connections working on ageing and the longevity economy. Founded in 2012, Aging2.0 strives to accelerate innovation to address the biggest challenges and opportunities in aging, with a community of 40k+ innovators across 31 countries. Their chapter network spans over 130 cities and has hosted more than 1000 events around the world. Oscar Zanutto (ISRAA) is the Treviso’s Aging 2.0 [13] Chapter Ambassador.

#### 2.2.4. Knowledge Platform Age-Friendly City the Hague

The Municipality of The Hague, interest groups, knowledge institutes, and social partners from the Hague region participate in the Knowledge Platform [14]. This initiative advises on the design of the integral monitor senior-friendliness and supports the recruitment of respondents. In addition, partners contribute relevant research and other information to the Knowledge Platform, so knowledge in the field of The Hague’s age-friendly city is further shared and enriched.

The several networks and stakeholder platforms described in this section have joined efforts to perform the mapping of social innovation ecosystems within the present study.

## 3. Materials and Methods

As referred in the introduction, the research methods to be applied in social innovation are still somehow immature and contested, and given the nature of the SHAFE network and its activities, it was opted to have an empirical study including quantitative methods, through the analysis of the closed survey (https://ec.europa.eu/eusurvey/runner/NET4Age-Friendly-Social-Innovation-Questionnaire (accessed on 27 October 2022) replies, combined with case study analysis, where practical examples of social innovation ecosystems were collected and grouped.

The research question underlying the study was: what are the number and nature of social innovation ecosystems working around the areas of SHAFE in Europe?

In order to achieve the reply to such question, the methods described were converted into practice through the following activities:

1—Development of the survey questions. The initial survey was developed by SHINE 2Europe and further commented and enriched by the co-authors. After several iterations, the semi-structured questionnaire developed encompassed a number of closed questions mainly aimed at characterising the social innovation ecosystem and the respondent’s organisation, added by a few open questions that are intended to collect the description of the social innovation initiatives and relevant links to be further showcased and disseminated in the future.

In terms of the data privacy policy, the survey was developed to allow for fully anonymised replies, however encompassing the possibility that the respondent wishes to be acknowledged and further contacted in relation to the initiative, and, in that case, the name, organisation, and email were collected.

2—Implementation of the survey online and short user testing. The final version of the questionnaire was transposed to an online tool to collect the replies. The tool selected was EUsurvey, the survey tool of the European Commission.

3—Dissemination of the survey among the Action Members and communication for the further dissemination through their networks. Once the survey was ready, it was disseminated through the COST Action CA19136 NET4Age-Friendly 490 members, as well as through the ECHAlliance, FABER, ISRAA, and SHAFE towards their members. A total of 61 valid replies were collected.

4—Analysis of the results. The survey replies were exported to Excel and further analysed in quantitative and qualitative terms. The quantitative results were mainly summarised in numbers and graphics, as the number of replies and the type of questions selected were not fit to provide a significant statistical analysis, which was, thus, not conducted. The qualitative inputs were gathered in tables, clustered under thematic groups, and further dissected in small explanatory texts.

## 4. Results

The survey collected 61 replies, mostly from NET4Age-Friendly members, from 28 different countries, as detailed in Table 1. It was disseminated through the networks involved and the participation was voluntary. As the respondents were not selected a priori, there was some imbalance in the country distribution, namely in what concerns members from Romania and Turkey, who demonstrated a very high participation.

From these, 14 wanted to remain anonymous, while the other 47 agreed to be acknowledged and have their contributions made public. In one of the latter cases, the name and organisation data were not provided, and one contribution was provided by a co-author.

### 4.1. Respondents

From a total of 61 responses, the majority reported being involved in the scientific and research world: 69% of the respondents (*n* = 42) work at a university or in other types of organisations dedicated to education and/or research. Hospital and care providers represent 10% (*n* = 6) of the respondents; public organisations and ecosystems/think tanks each represent 8% (*n* = 5).

Romania and Turkey were best represented among the respondents, respectively, 16% (*n* = 10) and 10% (*n* = 6). The vast majority of respondents were located in Europe, although the survey also received answers from Japan and Australia.

### 4.2. Ecosystems

It is relevant to highlight that 89% (*n* = 54) of the respondents think that their ecosystem is socially innovative. To contextualise the answer to this question, it is worth mentioning that the respondents were provided in the survey with the following quote and concepts to frame the subsequent replies:

According to the OECD “Social innovation refers to the design and implementation of new solutions that imply conceptual, process, product, or organisational change, which ultimately aim to improve the welfare and wellbeing of individuals and communities. Many initiatives undertaken by the social economy and by the civil society have proven to be innovative in dealing with socio-economic and environmental problems, while contributing to economic development. To fully tap the potential of social innovation, an enabling policy framework is needed to support public, non-profit and private actors to co-construct and implement socially innovative solutions and thereby contribute to address socio-economic issues, build stronger territorial resilience and better respond to future shocks” [15].

**Figure 1:**
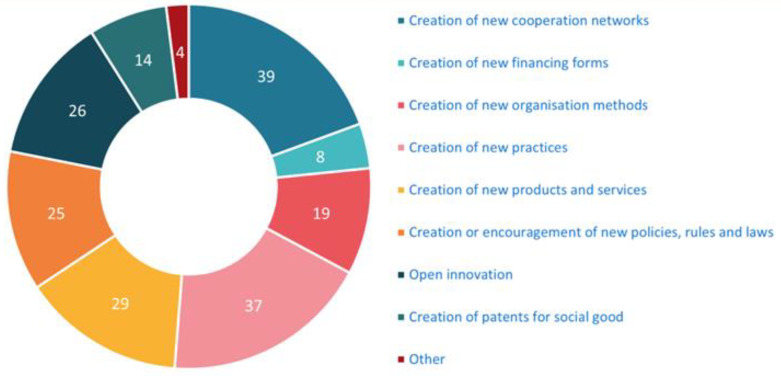
Types of social innovation developed in the respondent ecosystem.

On the question referring to which aspects of social innovation are developed, visible in Figure 1, the most given answers were “Creation of new cooperation networks” (*n* = 39) and “Creation of new practices (*n* = 37), followed by “Creation of new products and services” (*n* = 29), “Open innovation” (*n* = 26), and “Creation of encouragement of new policies, rules and law” (*n* = 25).

Slightly more than half of the ecosystems have a legal form (*n* = 35, 57%). Almost the same number of ecosystems have a brand (*n* = 34, 56%), which allows us to infer these actions as interconnected.

Ecosystems mainly consist of professionals/experts (*n* = 48), students (*n* = 44), citizens in general (*n* = 43), and business and industry (*n* = 33). It is very interesting to see a high number of citizens in general, as well as specific groups such as older people, women, and children—however, it is not clear yet if their role is proactive or whether they are in the ecosystems as recipients of innovation. Looking at the replies to question 9 of the survey, it is most probable that citizens still have a quite reactive/passive participation, and this is one aspect that will surely be relevant to explore in future works.

Most ecosystems consist of 0–9 organisations (67%, *n* = 41). Ten ecosystems consist of 10–19 organisations (16%, *n* = 10). Universities form the largest group of stakeholders of ecosystems (*n* = 54). Companies and businesses together are the second group (*n* = 41). Non-profit organisations and public administration follow as third and fourth (*n* = 26 and *n* = 25, respectively).

**Figure 2:**
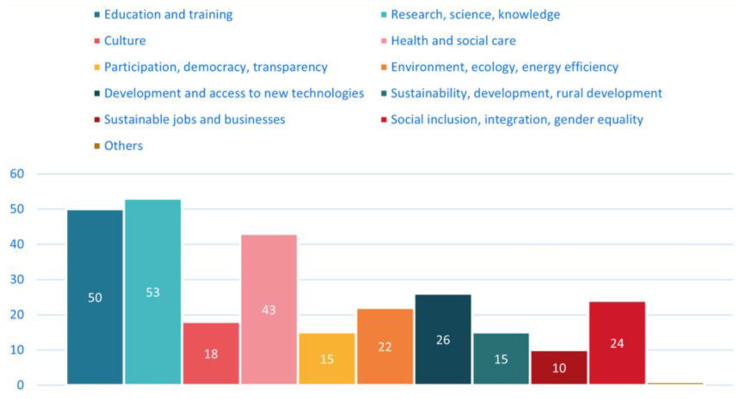
Areas of social innovation that the respondent ecosystem contributes to.

The area of research, science, and knowledge contributes most to social innovation (*n* = 53), followed by the areas of education and training (*n* = 50) and health and social care (*n* = 43), as visible in Figure 2. Development and access to new technologies (*n* = 26), and social inclusion, integration, and gender equality (*n* = 24) are also important parts of the work of ecosystems.

Ecosystems are mainly publicly funded (51%, *n* = 52). Own and private funds almost complete the picture of finances and ecosystems, with almost no innovative forms of funding reported.

A percentage of 59% (*n* = 36) of the ecosystems reach out to less than 1000 citizens. A reach of 1001–50,000 is achieved by 18 ecosystems (30%). The other 17 ecosystems have an adherence of over 50,000. Five of them even reach more than 1 million citizens.

Citizen participation in ecosystems is mainly realised by consultation with users/beneficiaries (*n* = 40), through forums, meetings without decision-making power (*n* = 36), and informative participation using standardised procedures (*n* = 32).

Transferability of ideas, processes, or models developed by the ecosystems is, thus far, only successful in 28 of the ecosystems (46%). Two ecosystems managed to transfer more than 100 new initiatives and seven ecosystems were successful in transferring 10–50 new initiatives. The other ecosystems indicated less transfers or did not provide an answer. Emerging from the ecosystems are mainly new associations or communities (*n* = 16), new companies or spin-offs (*n* = 14), new research organisations (*n* = 14), and new educational organisations (*n* = 13).

The interviewees pointed to some interesting initiatives that promote social innovation. Although most of the initiatives are cross-sectoral or involve various scientific areas, they were grouped into different clusters to gain a clearer overview of the areas that are more promoted or where it is possible to see more scientific and societal interest.

It is worth noting that half of the respondents reported that there has not been a reproduction or transfer of the ideas, processes, or models developed in their ecosystem to others. This implies that, despite various efforts on bringing the ecosystems together through the cross-border initiatives, networks, and international projects, many actors are still not connected to the wider global ecosystem. Health and technology are areas where many initiatives are being developed and, in many cases, these areas are interconnected: social innovative initiatives that, for example, aim at developing technology to improve people’s health.

The financial and business categories have the fewer initiatives pointed by our interviewees, demonstrating a clear gap where more investment and work are surely needed. It is also worth mentioning that many of the initiatives pointed out are funded projects that will come to an end. Presumably, it will be important to measure if the networks created during these projects persist after their end and what investment is taken for future sustainability of their outcomes.

## 5. Discussion

Considering the outcomes of the survey, some striking aspects emerge in relation to the introduction of policies aimed at and fostering processes of social innovation development, especially related to the overall profile that emerges from the analysis of the different ecosystems.

Composition: Universities together with research centres appear to be the main element characterising the structuring of ecosystems, together with the public-institutional component, only then followed by the private one. While this turns out to be natural and functional for paths to push innovation, there is also a need for institutions to broaden the base of informal stakeholders involved in moments of social transformation. Universities may consider including more participatory approaches in their research and expand their partnerships and agreements with grassroot organisation that allow them to have more bottom-up participation from citizens. Community-based participatory research (CPBR) approaches and their value in terms of community involvement and uptake and the use of results are well documented in the literature, and it is, thus, important to bring awareness to academics on these research areas.

Main outcomes: Looking at the predominant outcome of ecosystems, it appears that the creation of new practices, new services, and the stimulation of new rulemaking are among elements with the greatest social impact. New practices seem to arise from the networking of actors and their coordinated action within the framework, legitimised by belonging to the ecosystem. This means that the creation of social value is predominantly given by the liberation of the potential already present in the territory. Moreover, it appears that organisations devise and generate new contextual responses through mutual knowledge of activities, skills, and attitudes to social response, according to the type of needs demonstrated by the population.

Sizing: Most of the ecosystems are composed of up to ten organisations. This is a significant element to consider in order to expand the ecosystem presence and logic all over Europe. The lesson learnt is that a small group of local actors could have a closer interaction, having a direct connection with the needs and wants coming from the population. Furthermore, its small size allows it to act in an agile manner, being responsive to the opportunities that come also from the several funding strands, as public grants are reported as the most common vehicle to support social innovation.

Citizenship: Citizens are engaged in permanent ways and take part in local initiatives, on average, in fifty percent of the ecosystems, which means these are growing in direct connection with the local stakeholders involved.

Transferability: This is still a critical point. Only 28% of the ecosystems’ results and findings are exploited. More effort should be made in terms of presenting the solutions, methods, and interventions of social innovation towards other policymakers who could be in charge of understanding and adopting the “good practices” that come from other experiences. Once more, established local partnerships between academia and research organisations with non-profit associations and companies would be of interest to create solid and sustainable proposals for the stable adoption of social innovation processes and outcomes.

## 6. Conclusions

This empirical study developed and implemented a semi-structured survey for the identification of social innovation ecosystems among NET4Age-Friendly members and related local, regional, and national ecosystems, collecting 61 valid replies from 28 countries.

The analysis of the results helped to understand that the main drivers for social innovation are still rooted in academia—universities and research centres—and that this necessarily implies that the most common outputs relate to knowledge, science, and educational or training methods. Although this is an extremely important area, there is still the need to further invest in a more active engagement of other actors, such as NGOs, policymakers, and businesses, and especially, it is clear that citizen empowerment can be much improved.

The survey replies show that most of citizen engagement in social innovation is passive or reactive, meaning that the citizens simply reply to requests from other stakeholders, while social innovation processes would be much enriched if there was a proactive and creative approach from citizens to co-develop and enhance their own communities.

This aspect of citizen empowerment within NET4Age-Friendly and ways of increasing it at the local level will be further developed and taken for future works.

This study answers a gap in existing knowledge with a mapping of the social innovation initiatives and ecosystems working around SHAFE areas in Europe, which are not documented or understood. The broad significance of the study was the improvement in the understanding of the processes that need to be addressed in these ecosystems to promote more social innovation initiatives and support the implementation of the already achieved results.

The aspects related to the low levels of citizen engagement and empowerment and ways of increasing it at the local level will be further developed and taken for future works of NET4Age-Friendly.

One additional aspect for future research will be the geographical expansion of the research to other areas of the globe, namely America, Asia, and Australia, where social innovation experiences may enrich the portfolio collected in Europe and promote new knowledge development.

## Figures and Tables

**Table 1:** Distribution of survey replies by country.

Country	N. Respondents
Albania	1
Australia	1
Austria	1
Belarus	2
Belgium	1
Bulgaria	2
Croatia	2
Germany	3
Greece	2
Israel	1
Italy	4
Japan	1
Kosovo	1
Latvia	1
Netherlands	1
North Macedonia	1
Norway	4
Portugal	4
Moldova	1
Romania	10
Serbia	3
Slovenia	1
Spain	3
Sweden	1
Tunisia	1
Turkey	6
United Kingdom	1
Wales	1

## Data Availability

The data presented in this study are available on request from the corresponding author.
